# Consumer Wearables and the Integration of New Objective Measures in Oncology: Patient and Provider Perspectives

**DOI:** 10.2196/28664

**Published:** 2021-07-15

**Authors:** Lakshan N Fonseka, Benjamin KP Woo

**Affiliations:** 1 College of Osteopathic Medicine of the Pacific Western University of Health Sciences Pomona, CA United States; 2 Olive View-University of California, Los Angeles Medical Center Sylmar, CA United States

**Keywords:** consumer, wearables, smartwatch, cancer, oncology, chemotherapy, apps

## Abstract

With one in five adults in the United States owning a smartwatch or fitness tracker, these devices are poised to impact all aspects of medicine by offering a more objective approach to replace self-reported data. Oncology has proved to be a prototypical example, and wearables offer immediate benefits to patients and oncologists with the ability to track symptoms and health metrics in real time. We aimed to review the recent literature on consumer-grade wearables and its current applications in cancer from the perspective of both the patient and the provider. The relevant studies suggested that these devices offer benefits, such as improved medication adherence and accuracy of symptom tracking over self-reported data, as well as insights that increase patient empowerment. Physical activity is consistently correlated with stronger patient outcomes, and a patient’s real-time metrics were found to be capable of tracking medication side effects and toxicity. Studies have made associations between wearable data and telomere shortening, cardiovascular disease, alcohol consumption, sleep apnea, and other conditions. The objective data obtained by the wearable presents a more complete picture of an individual’s health than the snapshot of a 15-minute office visit and a single set of vital signs. Real-time metrics can be translated into a digital phenotype that identifies risk factors specific to each patient, and shared risk factors across one’s social network may uncover common environmental exposures detrimental to one’s health. Wearable data and its upcoming integration with social media will be the foundation for the next generation of personalized medicine.

## Introduction

In 2020, one in five adults in the United States wore a smartwatch or similar fitness tracker [1]. As its prevalence increases, its integration in medical management and research increases as well. Wearables have introduced objective measures to replace self-reported data and supply new variables that offer previously unattainable insights. With increasing options and entry points for consumer wearables, the prevalence of devices, such as the Apple Watch, Fitbit, Samsung Galaxy Watch, and others, is increasing. These consumer wearables are poised to have an impact across all aspects of medicine, and oncology has proved to be a prototypical example.

Wearable research in oncology applications has demonstrated benefits, such as improved medication adherence and accuracy of symptom tracking, over self-reported data, as well as insights that increase patient empowerment. Physical activity is consistently correlated with stronger patient outcomes, and a patient’s real-time metrics were found to be capable of tracking medication side effects and toxicity. Studies have made associations between wearable data and telomere shortening, cardiovascular disease, alcohol consumption, sleep apnea, and other conditions. Herein, we review the literature on the new objective measures introduced by consumer-grade wearables and the current applications in cancer from the perspectives of both the patient and the provider.

### Objective Measures

The advent of wearables brings new objective measures along with the ability to remotely monitor the data being collected in real time. Before delving into the oncological applications, we summarize the most prevalent wearable measures as an introduction to subsequent studies. The accuracy of these measures, however, remains mostly untested. Some consumers have reported perceived inaccuracy in step counts and sleep metrics in Amazon product reviews [2]. The metrics have also been shown to be affected by a slower gait [3]. Despite this potential limitation to its objectivity, consumer wearables have shown validity in tracking physical activity and are a step forward from self-reported activity data [4].

Of these new measures, physical activity is often the most commonly used and is usually calculated by consumer devices as steps or calories per day. Still, as Beauchamp et al highlights through 25 studies, there exists widespread diversity in metrics gathered. For example, physical activity may be measured as not only steps or calories per day, but also steps or calories per hour, sedentary minutes, standing time, etc. Sleep information is commonly collected, though this also is reported by differing metrics including sleep efficiency percentage, sleep hours, nighttime total wake time, time in bed, and number of awakenings [5].

Many consumer wearables promote continuous heart rate measurement and heart rate variability, with the Apple Watch expanding the concept a step further with a single-lead electrocardiogram (ECG) in certain models. As will be detailed further in this article, the focus of the single-lead ECG is atrial fibrillation detection, but it may also be helpful in QT prolongation detection.

Together, physical activity, sleep, and heart rate tracking form the core of consumer wearable measures. Other features include fall detection, handwashing, and preliminary blood oxygen saturation. Many more exciting new measures are upcoming and are mentioned in the discussion on future outlook.

## Impact on Oncology Care

### Patient Perspective

#### Apps

A search for “cancer” in any app store reveals dedicated services aimed to support cancer patients. There is an increasing number of apps that take advantage of the typical wrist location of the wearable to send essential alerts to the patient, such as timely medication reminders to increase adherence. One such app is the Medopad chemotherapy app, which provides on-wrist medication reminders, but also allows patients to submit symptoms and temperatures [6]. The LivingWith: Cancer Support app by Pfizer maintains the medication reminder system and adds the ability to track self-reported mood and pain in just a few taps.

One of the most interesting features comes from the chemoWave app, which maintains the symptom tracking and medication logging features, and combines them with wearable data to generate insights in an easily understandable manner. For example, the app may provide the insight that on days with less sleep, the patient is 43% more likely to experience a headache [7]. The app uses artificial intelligence to generate “personal insights” as in the above example, as well as “global insights” such as “on days chemoWave users like you took more than 7000 steps, they were 90% more likely to feel better” [8]. This appears to be the first consumer-level integration of wearable data with wearable-tracked activity. chemoWave allows users to generate graphs that detail symptoms, exercise, medications, water intake, and more. Patients can show these graphs to the oncologist and assisting doctors to pinpoint changes in patient activity with associated medications or procedures. The benefits include increased accuracy compared with self-reported data, such as having a patient in the office and prompting them to remember what side effects they experienced on days 3 or 4 of treatment. Oncologists have used chemoWave to view trends and better time a patient’s next cycle of chemotherapy. Communication is also streamlined, as patients are able to send doctors daily updates through the app [9].

Although not directly related to cancer, the most highly reviewed app that appears when searching for “cancer” in the Apple App Store is Motivation-Daily Quotes, with over 444,000 reviews at an average rating of 4.8/5. Reviews on the Apple App Store include comments, such as “this app helped me to get through so many tough times,” and follow a general theme of appreciating positivity from the app. Users seem to particularly enjoy the app’s reminder feature. As one user says, “whenever I need encouragement to continue living my life and keep a positive mindset, the app throws the perfect quote for the situation in the perfect moment” [10]. It may not utilize the wearable’s data collection features, but the app takes advantage of the proximity and direct interactivity of the wearable to provide consistent reminders that over time may improve mood and adherence.

Some of the aforementioned apps allow users to connect with family members and cancer support groups to further tap into the motivational aspect. Though functional, these apps are not always successful. For instance, reviews on the Pfizer app mention a “clunky interface,” and users have specifically reported choosing to create a group chat on Facebook instead. Recently, Facebook announced the development of a smartwatch intended to connect users with health apps, which is expected to release in 2022 [11]. Patients who typically receive social support over Facebook, as with the patient who reviewed the Pfizer app, may opt for this smartwatch. At a minimum, social support received through Facebook is likely to be more often seen by the patient through the smartwatch and may augment the overall perception of support to a greater degree. Though only speculation, it is likely that Facebook would incorporate sharing of tracked measures, such as step counts, which may increase the timeliness of support messages by alerting the patient’s support network when physical activity has decreased persistently. In this way, the wearable would allow a patient’s supporters to have greater involvement and interactivity in the patient’s overall health, which may contribute to increased morale for the patient and respective social network.

#### Geriatrics

As cancer remains a significant disease affecting the geriatric population, it is necessary to consider the relevant applications and ease of use [12,13]. Less than half of adults over the age of 65 years reported owning a smartphone in 2017, with an increase of 24% from 2013. Even so, up to two-thirds of adults over the age of 65 years say they go online [14]. Thus, there may be a subset of older adults who do not have a smartphone, but use other devices to go online [15]. In addition, Hoogland et al showed that despite feeling less confident in using technology, older adults with cancer were mostly agreeable to using health information technology to communicate with their oncology care team [16]. Yet, even if older adults were open to wearables, most consumer devices require a connected smartphone to function as intended. The lack of a smartphone therefore becomes a significant deterrent to wearable use. The Facebook smartwatch will be independent of any smartphone, perhaps offering ease of use to elderly patients without a smartphone. Another challenge identified in elderly populations is lack of internet access, with one out of three patients without any connection to home internet [14]. The Facebook smartwatch will offer its own data connection, further extending coverage of elderly patients and allowing the benefits of wearables to reach geriatric cancer patients.

#### Young Adults

On the other end of the age spectrum, cancer survivors aged 18 years or older tend to struggle with meeting physical activity recommendations intended to reduce cancer recurrence, mortality, and adverse chronic conditions. These guidelines consist of two strength training sessions per week with either 75 minutes of vigorous physical activity or 150 minutes of moderate activity per week [17]. Miropolsky et al tested the use of a Fitbit device with 13 young adult cancer survivors and found that the participants reported the Fitbit device helped them to reach activity goals. However, this study implemented a buddy system, in which the cancer survivors chose a friend to participate in reaching activity goals. Though participants reported that both the Fitbit device and the buddy system were significant motivators, the impact of the Fitbit device alone is unclear. Nonetheless, this study shows that consumer wearables are a feasible option that may help to increase physical activity in young adult cancer survivors [18].

#### Melanoma

Up to this point, the information has been relevant for all cancers, but the fifth most common cancer, melanoma, presents a unique opportunity for wearables, as the foremost modifiable risk factor is an individual’s UV exposure [19,20]. Although no smartwatches or Fitbit devices can directly measure UV radiation, a new commercially available wearable called Shade contains a UV sensor and provides users with a timeframe of how long they can safely be under the sun. Notifications can be sent through the on-wrist device and companion app every time a user comes 20% closer to the UV exposure limit [21]. A randomized controlled trial involving the use of Shade in melanoma survivors is underway, with results expected to be available in 2022 [22]. Nagelhout et al investigated the feasibility of the Shade wearable and found that 73% of adults and 61% of children selected that they wore the device “all of the time they were outside” on a questionnaire following a 2-week period with Shade [23]. The largest obstacle may be the cost at US $499, but nevertheless, the first commercially available UV sensor is a milestone in consumer wearables and may be invaluable to patients with a family or personal history of melanoma to assuage concerns of sun exposure.

### Provider Perspective

From the viewpoint of the provider, the focus shifts onto the role of wearables in the management of patients currently undergoing cancer treatment, as well as cancer survivors. Numerous studies have been conducted on this topic, but the most recent and relevant studies that used typical consumer-level devices or measures will be briefly summarized. The goal of this section is not to analyze the directions of future clinical trials using wearables, but rather to update clinicians on how a patient’s wearable may provide clinically relevant data in the near future.

#### Physical Activity

Several studies have shown that physical activity tracked by wearables is a strong predictor of outcomes in cancer patients [24]. In patients undergoing chemotherapy or radiation treatment, lower step counts were found to be correlated with lower quality of life, greater hospitalization risk, decreased adherence, depression, and shorter survival [25-28]. On the other hand, higher step counts were associated with decreased postoperative complications in abdominal cancer patients and better functional status with reduced likelihood of hospitalization or death [29,30]. A patient, family member, or clinician can generate a graph of the patient’s step counts before, during, and after treatment using the wearable’s specific app or one of the previously discussed apps. This may aid in identifying at-risk patients and provides another tool to consider in prognosis, especially if done remotely. In addition to tracking physical activity, wearables have been shown to increase physical activity among cancer patients and survivors as well [17,31,32]. Conversely, Rahimy et al found that a Fitbit program for survivors of endometrial cancer led to a transient 22% increase in average steps at 6 months, but by the end of the study at 9 months, participants had returned to their baseline activity levels with no change in BMI. The authors interestingly found step count to be correlated with emotional well-being (*P*=.005), though this result may represent that subjects who are physically capable of higher step counts are more likely to be healthy, with less severe residual effects of disease and therefore an improved emotional well-being [33]. Overall, these studies suggest that while wearable fitness trackers may motivate some patients, others may require different interventions to inspire enduring lifestyle changes.

#### Chemotherapy Toxicity

Consumer wearable data can be further used to assess chemotherapy toxicity and side effects using step count, heart rate, and ECG data. In a pilot study, chemotherapy patients had their step counts tracked by a smartphone with a pedometer app. The study found that contacting patients if step counts decreased by 15% or more helped to identify chemotherapy toxicity [34]. While virtually all consumer wearables track step count, it is commonplace to also see wearables that can track heart rate. However, only relatively newer models of the Apple Watch are able to record a single-lead ECG. Both heart rate and ECG data may be used to track side effects in patients taking cardiotoxic chemotherapy drugs. For example, taxanes and angiogenesis inhibitors, such as thalidomide, can lead to sinus bradycardia [35]. Smartwatches can detect bradycardia, and some devices, such as the Apple Watch, allow a threshold heart rate to be set, such that the patient is notified if the heart rate falls below a specified value. Physicians can instruct patients on what heart rate to specify for the alert and thereby have access to continuous remote monitoring for bradycardia.

Atrial fibrillation is a common side effect of chemotherapy regimens that include anthracyclines, trastuzumab, busulfan, and cyclophosphamide [36]. ECG data has the potential to detect atrial fibrillation, though there is conflicting data as to how often false positives occur [36-38]. QT interval prolongation may also be tracked. Timely detection of QT prolongation can prevent conversion to torsades-de-pointes, a life-threatening ventricular tachycardia. Studies have previously approved nonconsumer wearables, such as KardiaMobile-6L, to monitor the QT interval in COVID-19 patients, as remote monitoring became a sudden essential need during the COVID-19 pandemic [39]. Regarding consumer-level wearables, the American Cancer Society recommends that patients who are starting chemotherapy drugs associated with QT prolongation should obtain a baseline 12-lead ECG, but subsequent monitoring can be “accurately and safely performed with topical devices” such as the Apple Watch [40].

#### Digital Phenotype

Ultimately, wearables have the potential to create a digital phenotype of each patient, which is a real-time record of patient metrics that are translated into stress levels, risk factors, and other determinants of health [41,42]. This potential was illustrated by Teo et al, where self-reported demographics, socioeconomic status, and lifestyle factors were compared with Fitbit-recorded total sleep time (the amount of time a person is asleep) and sleep efficiency (the fraction of time spent asleep over the time a person is in bed) in 482 individuals. The total sleep time was found to be associated with age, gender, habitual alcohol consumption, ethnicity, and occupation type. Interestingly, the authors also sequenced the DNA of study participants and used quantitative polymerase chain reaction to estimate telomere length, a biomarker of aging. Users with insufficient sleep were more likely to show premature telomere shortening. Subjects who had adequate sleep (7 hours or more) had telomeres that were 356 base pairs longer on average than those with insufficient sleep (less than 5 hours) (*P*=.02). In addition, sleep efficiency was associated with cardiovascular disease risk markers such as BMI. The authors compared the same associations with self-reported sleep data, and no significant links were found, emphasizing the overall improvement in data collection with wearables rather than self-reported information [42].

Furthermore, Tison et al used deep learning algorithms that showed promise in developing digital biomarkers for screening sleep apnea and hypertension with an accuracy of 90% and 82%, respectively [43]. These studies further support the role of wearable data in ushering in a new era of personalized medicine, especially in oncology. The massive inflow of wearable data can be integrated into a straightforward digital phenotype, enabling clinicians to assess in real time a patient’s baseline risk factors and any changes that may occur as a result of disease progression or a side effect of a newly initiated chemotherapy regimen. A digital phenotype may also guide interventions, as clinicians can avoid starting a patient on medications with side effects that can deleteriously interact with a patient’s risk factors (ie, a cardiotoxic drug in a patient whose digital phenotype shows significant cardiovascular risk).

## Future Directions and Conclusions

The impact of wearables has seeped into all fields of research and poses unique opportunities previously unseen in oncology. The above studies indicate clear benefits to both patients and providers. Based on the presented literature, oncologists should use wearable data to increase treatment adherence and patient-physician communication, as well as monitor physical activity and ECG data to guide medical decision-making. The objective measures introduced by consumer wearables will soon be joined by others, including oxygen saturation, blood glucose, and biomarkers in sweat that may indicate cortisol levels [44,45]. Updates to existing consumer devices will include advancements to ECGs, the ability to detect smoking gestures, and smartwatch UV radiation sensors [24]. Completely new wearables will enter the consumer market, such as a headband for the detection of glioblastoma [46].

Soon to be uncovered is how the merging of social media and wearables will expand the landscape. With the imminent launch of the Facebook smartwatch, social media will be integrated with wearable data. Thus, oncologists will have the data of not only their patients, but also each patient’s peers. This will allow the oncologist to individualize treatment plans to address the specific risk factors of the patient. For example, location data of a patient and members of the patient’s social network may yield data on healthy food access, neighborhood crime rates, air quality, socioeconomic status, local policies on marijuana use, etc, which can serve as predictors of an individual’s health in conjunction with wearable data such as physical activity and total sleep time [47]. Likewise, any regional exposures, such as air pollution and lead levels with wearable-measured disease states (ie, reduced physical activity and increased time at hospitals), may unveil carcinogenic environmental exposures [48]. The integration of social media will contribute to the development of future wearable-derived health predictors and assist oncologists in tailoring treatment to target risk factors specific to each patient.

Wearables have become increasingly prevalent in the consumer market, and a look in any direction is likely to reveal a Fitbit, a smartwatch, or another wearable on someone’s wrist. From the patient’s perspective, consumer wearables allow for improved medication adherence, symptom tracking and insights, communication with physicians and family members, motivational support, and increased reach to patients who were previously inaccessible despite the advent of the smartphone. From the provider’s perspective, wearables assist in achieving the benefits of continuous rather than episodic care, especially in monitoring for medication side effects and toxicity. Oncologists are able to track changes in a patient’s baseline metrics outside of the office and infer if any recent treatment changes were responsible. Real-time metrics can be translated into a digital phenotype that identifies risk factors specific to each patient, and shared risk factors across one’s social network may uncover common environmental exposures detrimental to one’s health. The objective data obtained by the wearable, viewed in the context of one’s social network, presents a more complete picture of an individual’s health than the snapshot of a 15-minute office visit and a single set of vital signs. Overall, wearable data and its upcoming integration with social media will be the foundation for the next generation of personalized medicine.

## Abbreviations

ECG: electrocardiogram
